# Insights into the Mitochondrial and Nuclear Genome Diversity of Two High Yielding Strains of Laying Hens

**DOI:** 10.3390/ani11030825

**Published:** 2021-03-15

**Authors:** Clara Heumann-Kiesler, Vera Sommerfeld, Hanna Iffland, Jörn Bennewitz, Markus Rodehutscord, Martin Hasselmann

**Affiliations:** 1 Department of Livestock Population Genomics, Institute of Animal Science, University of Hohenheim, 70599 Stuttgart, Germany; martin.hasselmann@uni-hohenheim.de; 2 Department of Animal Nutrition, Institute of Animal Science, University of Hohenheim, 70599 Stuttgart, Germany; v.sommerfeld@uni-hohenheim.de (V.S.); markus.rodehutscord@uni-hohenheim.de (M.R.); 3 Department of Animal Genetics and Breeding, Institute of Animal Science, University of Hohenheim, 70599 Stuttgart, Germany; hannah.iffland@uni-hohenheim.de (H.I.); j.bennewitz@uni-hohenheim.de (J.B.)

**Keywords:** haplotype, phosphorus utilization, body weight, genetic diversity, relatedness, population structure, genotyping

## Abstract

**Simple Summary:**

Mitochondria are commonly known as “the powerhouse of the cell”, influencing the fitness, lifespan and metabolism of eukaryotic organisms. In our study we examined mitochondrial and nuclear genomic diversity in two high yielding strains of laying hens. We tested if the mitochondrial genome affects functional traits such as body weight and phosphorus utilization. We discovered a surprisingly low mitochondrial genetic diversity and an unequal distribution of the haplotypes among both strains, leading to limitations of robust links to phenotypic traits. In contrast, we found similar levels of nuclear genome diversity in both strains. Our study explores the potential influence of the mitochondrial genome on phenotypic traits and thus contributes to a better understanding of the function of this organelle in laying hens. Further, we focus on its usefulness as a genetic marker, which is often underestimated in breeding approaches, given the different inheritance mechanism compared to the nuclear genome.

**Abstract:**

Mitochondria are essential components of eukaryotes as they are involved in several organismic key processes such as energy production, apoptosis and cell growth. Despite their importance for the metabolism and physiology of all eukaryotic organisms, the impact of mitochondrial haplotype variation has only been studied for very few species. In this study we sequenced the mitochondrial genome of 180 individuals from two different strains of laying hens. The resulting haplotypes were combined with performance data such as body weight, feed intake and phosphorus utilization to assess their influence on the hens in five different life stages. After detecting a surprisingly low level of genetic diversity, we investigated the nuclear genetic background to estimate whether the low mitochondrial diversity is representative for the whole genetic background of the strains. Our results highlight the need for more in-depth investigation of the genetic compositions and mito-nuclear interaction in individuals to elucidate the basis of phenotypic performance differences. In addition, we raise the question of how the lack of mitochondrial variation developed, since the mitochondrial genome represents genetic information usually not considered in breeding approaches.

## 1. Introduction

The domestic chicken is the most popular and widely distributed domestic fowl worldwide [1] and hence it is a stable source of protein, including meat and eggs [2]. In contrast to the advances made by high-throughput nuclear genotype analyses in chicken [3,4], the insights into mitochondrial (mt) genome diversity are rather sparse. For many animals it is known that mt genome diversity can have a remarkable impact on a variety of traits, such as meat quality in pigs [5] and the metabolic capacity of dairy cows [6]. In chickens, previous studies have shown that mutations in the mitochondrial genome can have strong physiological effects. This includes the adaptation to high altitudes of the Tibetan chicken [7,8] and economically important traits such as egg quality and body weight [9]. Even though the whole mitochondrial genome has been used in many studies of domesticated animals such as goats [10], horses [11] and pigs [12], most studies of the chicken relied on partial mitochondrial sequences or the protein coding sequences [1] and thus, studies covering the whole mt genome are underrepresented. Given the importance of the genetic background on organismic physiological performance, beside the nuclear genome, the in-depth study of mitochondrial genomes and their variation are of utmost interest.

Mitochondria are commonly known as the powerhouse of the cell since they contribute to the energy metabolism by generating ATP [13]. During the process of oxidative phosphorylation (OXPHOS) they are a major producer of reactive oxygen species (ROS) and thus contribute to oxidative stress [14]. Therefore, the mitochondrial energy metabolism is directly linked to the availability of phosphorus (P).

Additional to their role as the cellular energy supplier, mitochondria contribute to several other key cellular processes such as the biosynthesis of important macromolecules [13], calcium (Ca) homeostasis [15] and ageing [16] that have been mainly studied within genetic model organisms such as fruit flies or mice [17,18].

Mitochondria carry their own genome, which in chickens is approximately 16 kb long and contains 13 protein coding genes, 22 tRNA genes, two rRNA genes and one regulatory control region [19]. Due to their maternal inheritance, simple molecular structure [1] and relative high copy number [20] they traditionally serve as popular genetic markers in molecular biology [21].

As all living organisms, chickens need P for their growth, health and energy metabolism [22]. For chickens, P in plant-based feedstuff is mainly present as phytic-acid (InsP_6_) [23,24], which consists of six phosphate groups and one *myo*-inositol ring. While passing through the digestive tract, stepwise InsP_6_ dephosphorylation results in the release of phosphate and *myo*-inositol [25]. However, the ability of poultry to degrade InsP_6_ is limited. Feed supplements produced from rock phosphates maintain the P supply. The availability of rock phosphate is finite [26], which makes reducing its use one of the major challenges in food production.

To improve P utilization efficiency, we need to understand the genetic and nongenetic background for the processes influencing the formation of inositol derivates during InsP_6_ degradation and their relevance for P utilization. It is furthermore important to understand how these processes change over time, since the P demand changes during a chicken’s lifetime, depending on factors such as growth or egg laying [27].

We aim to understand the mitochondrial genetic variation in laying hens and hypothesize that different haplotypes lead to physiologically distinct phenotypes. To study this impact, we obtained whole mitochondrial genomes from a large experimental setup of laying hens that had the abovementioned aspects, P and Ca utilization and phytate degradation in focus (Sommerfeld 2020a, 2020b [27,28]). We analyse the body weight and feed intake and hypothesize that the performance of individuals with different mitochondrial haplotypes differs within and between production periods according to the animals’ needs. Thus, we can gain insights into the connection between the genetic variation of mitochondrial genomes and their physiological effects and contribute to a better understanding of these effects in laying hens. We enriched our data by nuclear genetic information of a subset of individuals that enable us to gain important insights into the nuclear genetic structure of the population and potential effects of nuclear and mitochondrial interactions [29,30,31].

## 2. Materials and Methods

### 2.1. Animal Experiments

The animal experiments were performed at the Agricultural Experiment Station of the University of Hohenheim, Germany. They were approved by the Regierungspräsidium Tübingen, Germany (Project no. HOH50/17TE) in accordance with the German Animal Welfare Legislation.

We used 180 laying hens: 90 brown (Lohmann Brown Classic) and 90 white (Lohmann LSL-Classic) leghorn hybrids obtained from Lohmann Tierzucht GmbH (Cuxhaven, Germany). The hens originated from two experiments addressing the changes of utilization of P and Ca in different periods of the hens’ life (production periods, experiment 1) and under adequate or reduced P and Ca supply during the peak of egg production (experiment 2). The first experiment provided 100 individuals and is described in detail in Sommerfeld et al. 2020a [27]; the second one provided 80 animals and is described in Sommerfeld et al. 2020b [28]. We briefly summarize them in the following.

**Figure 1:**
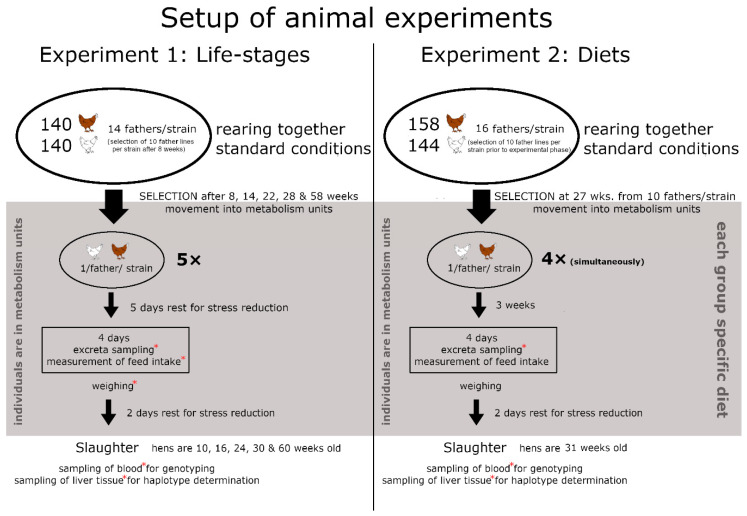
Schematic description of the setup of both animal experiments. Red asterisks mark material and measurements that were used in this study.

In experiment 1 (Figure 1), the animals were raised as one group in floor pens on deep litter bedding, with diets according to the specific requirements in each period based on soybean and corn meal, but no difference to the recommendations as given in detail within Table 1 in Sommerfeld et al. 2020a [27]. After 8, 14, 22, 28, and 58 weeks 10 hens per father and strain were chosen and moved into the metabolism units (1 m × 1 m × 1 m, arranged in a randomized block design, where 2 units formed one block, each metabolism unit contained one individual) where after five days of rest excreta and feed intake was measured for four days for each individual. Feed was available for ad libitum consumption and no changes in the diet were made. The animals were weighed and after two days of rest the hens were killed by decapitation following anaesthesia using a gas mixture [32] and samples of blood and liver tissue were taken, thus the hens were 10, 16, 24, 30, and 60 weeks old. In the following the five sampling points will be named as period 1 to period 5.

For experiment 2 (Figure 1), the animals were raised together under the same conditions as in experiment 1. After 27 weeks hens from 20 fathers (10 per strain) were separated into four groups in which each group contained one hen per father and the hens were placed into the metabolism units (1 m × 1 m × 1 m, arranged in a randomized block design, where 8 units formed one block and each metabolism unit contained one individual). For three weeks the individuals received feed for ad libitum consumption with specific diets for each group based on soybean and corn meal, containing recommended or lower concentrations of Ca and P (Table 1). The excreta of each hen were collected and the feed intake was monitored for four days in week 30. The hens were killed at 31 weeks of age (peak of egg production). As in experiment 1 blood and liver tissue were sampled.

### 2.2. DNA Samples and Extraction

We extracted DNA twice per animal: from liver tissue for the haplotype analyses and from blood for genotyping. For the haplotype reconstruction all 180 individuals were used, while for the nuclear genotyping 52 samples from individuals of the first experiment (26 brown and 26 white, covering period 1 to period 4) and 55 from the second experiment (27 brown and 28 white, covering all four diets) were included. The animals for the genotyping were selected randomly; care was taken to include an even amount of brown and white animals.

Liver samples were taken after slaughtering as described above and directly transferred to dry ice. We extracted high molecular DNA from frozen tissue using the DNeasy Blood & Tissue Kit (Qiagen, Hilden, Germany) following the purification of total DNA from Animal Tissues protocol.

For genotyping, the DNA from 53 brown and 54 white hens was extracted from blood which was directly taken at the sampling sessions as described above, stored in EDTA and further processed using the Maxwell 16 Blood DNA Purification Kit on a Maxwell 16 MDx instrument (Promega, Madison, WI, USA).

### 2.3. Analysis of Mitochondrial Haplotypes

#### 2.3.1. Long Range PCR and Next Generation Amplicon Sequencing

The mt genome was individually amplified by two overlapping fragments of 8935 bp and 9522 bp length using a Long Range (LR) PCR Kit (Qiagen, Hilden, Germany). Primers were designed and tested if they amplify other fragments of the same size using Primer BLAST [33]. Primer sequences for fragment 1 are: F: ACGCCACAGCTAAAACCCCCAGC R: TGGATGGTGGAGAGGCGGTTGT and fragment 2: F: TCCTGCTTGCCCTCCCCTCCCT R: CGCACCCGCACTGTGAAGGGCAA. The reactions were set up according to the protocol of the manufacturer with primer annealing temperatures of 63 °C for fragment 1 and 60 °C for fragment 2. The elongation phase was set to 9 min for both fragments. Successful amplifications were verified via gel electrophoresis on a 1% agarose gel (VWR International GmbH, Darmstadt, Germany) stained with gel-red (Biotium Inc., Fremont, CA, USA). The PCR products were precipitated with ethanol; quality and quantity of the products were measured using a NanoDrop 2000/2000c Spectrophotometer and Qubit Fluorometer (Thermo Fisher Scientific Inc., Waltham MA, USA). Resulting purified fragments were equimolarly pooled for sequencing. The samples were sequenced by CeGaT (CeGaT GmbH, Tübingen, Germany), using the Nextera XT Kit (Illumina Inc., San Diego, CA, USA) for library preparation, on an Illumina NovaSeq 6000 MiSeq expecting 150 bp paired-end reads.

#### 2.3.2. Sequence Analysis

The sequencing reads were demultiplexed and adapter-trimmed by CeGaT using Illumina bclfastq (v2.20; Illumina Inc., San Diego, CA, USA) and Skewer (Version 0.2.2 [34]), followed by a quality-check in FastQC [35] of files containing comparatively high and low numbers of reads. After the removal of primer sequences with Cutadapt (version 2.5 [36]) the reads were quality-trimmed in Trimmomatic (version 0.32 [37]) using the following parameters: SLIDINGWINDOW:4:15 MINLEN:50 CROP:148. The used settings cut the read if the average quality of 4 bases is below 15, discard reads which are shorter than 50 bases and cut the reads that are longer than 148 bases.

We mapped the reads against a published white leghorn mitochondrial genome (AP003317 [38]) using the algorithm implemented in Geneious Prime (version 2020.0.2 https://www.geneious.com, accessed on 5 November 2019). Duplicates were removed before mapping using the Dedupe Duplicate Read Remover 38.37 by Brian Bushnell as implemented in Geneious Prime using default parameters, as well as the variant call with default settings except for minimum coverage, which was set to five. The resulting output was then used to create consensus sequences using GATK FastaAlternateReferenceMaker [39].

The resulting consensus sequences included a poly-C site with 8 to 13 Cytosins (position 3940 and following). The site did not only vary in its length without a pattern between individuals but also within the reads of most individuals. The issue could not be resolved as in both sequencing approaches (Illumina and Sanger-technology) these sites led to read termination. Consequently, we followed the most conserved approach and restricted the length down to 8 Cytosins for all individuals.

For those individuals that showed coverage close to zero in one of the two PCR fragments, the missing parts were sequenced using a PCR based Sanger-sequencing approach. A list of the corresponding individuals, used primers, reagents and PCR protocol can be found in Tables S1–S3, Supplementary Materials. Sanger-sequencing reactions were performed by Microsynth AG (Balgach, Switzerland) and the resulting sequences were edited in Geneious Prime.

#### 2.3.3. Validation of Sequencing Results

To approve the resulting consensus sequences, the primer- and quality-trimmed reads were additionally mapped against a more distant mt genome to the leghorn variant (*Gallus gallus*, NCBI Accession AP003322, [40]) using the same parameters as described above.

To exclude LR-PCR biased artefacts, we resequenced the whole mt genome of one individual with Sanger-technology using mt DNA and a set of 22 oligonucleotides that result in 22 overlapping fragments. Furthermore, the long-range PCR fragments that were used for the library construction were Sanger sequenced using the same set of oligonucleotides (Supplementary Table S1).

#### 2.3.4. Alignment and Haplotype Reconstruction

The consensus sequences were aligned using the implemented algorithm in Geneious Prime with default settings.

Haplotype networks were generated based on the alignment using the TCS algorithm [41] implemented in PopART v. 1.7 [42]. Additionally, a phylogenetic analysis was conducted via Maximum Likelihood, for which we initially identified the nucleotide substitution model that best fit to the data, based on the program Model Test implemented in MEGA X [43]. Based on the lowest Bayesian Information Criterion (BIC) score, the Hasegawa–Kishino–Yano model [44] was chosen for tree construction with 500 bootstrap replicates and default settings in MEGA X.

### 2.4. Analysis of Nuclear Genotype Data

After the identification of the mt haplotypes, we aimed to gain additional insights into the nuclear genome diversity and by analysing nuclear genotype data obtained with the Illumina 60K chicken Infinium iSelect chip (Illumina Inc., San Diego, CA, USA). For the following analyses we used 54 white and 53 brown individuals from both experiments.

#### 2.4.1. Filtering

The resulting SNPs were filtered for cluster separation ≥0.4 in the GenomeStudio software (v. 2011.1, Illumina Inc., San Diego, CA, USA) and then exported for downstream analyses. Prior to the next filtering steps, the SNP positions were transferred to the newest chicken genome version (GRCg6a, Accession: GCF_000002315.6) and the two strains were separated using vcftools [45]. The SNPs were filtered in vcftools for missingness (95%), minor allele count (3) and minor allele frequency (0.03) prior to further analysis. For the population structure analysis with ADMIXTURE [46] Linkage disequilibrium (LD) pruning was performed after filtering as recommended using PLINK version 1.9 [47], where all SNPs with a R^2^ value greater than 0.1 with any other SNP within a 50-SNP sliding window (advanced by 10 SNPs each time) is removed as described in the manual. The dataset was filtered without previous separation of the strains for additional ADMIXTURE analyses as described above.

#### 2.4.2. Analysis of Nuclear Genetic Diversity

To gain insights into the nuclear genome diversity and relationships between individuals within the strains, a genomic relationship matrix (G-Matrix) was performed with the *AGHmatrix* [48] package in R (R Core Team 2020, Version 3.0.3) using the VanRaden method [49]. Note that the G-Matrix is usually used to study complex traits, but was used here to obtain some insight of genomic relationships. The two strains were analyzed separately, since we look at them as two distinct lines. After the calculation of the matrices, statistical analyses were performed in JMP Pro (Version 13. SAS Institute Inc., Cary, NC, USA, 1989–2019) to detect the impact of the paternal and possible maternal background and differences between the strains and mt haplotypes. The data was tested for normality using Shapiro–Wilk test. Wilcoxon/Kruskal–Wallis tests were used for the comparison of two groups and Steel–Dwass tests for multiple comparisons.

The differentiation between both strains was estimated using Weir and Cockerham’s F-statistics fixation-index (F_ST_) [50] as implemented in vcftools based on the dataset including both strains filtered together. To analyze the differentiation between the mt haplotypes the F_ST_ between the different haplotypes was estimated the same way based on the file only containing the brown strain.

To estimate population structure ADMIXTURE was used on (1) the merged data set obtained from both strains, and (2) separate data sets for each strain, to gain deeper insights into the strain structure. The number of ancestral populations (K) was set from 2 to 6 for all runs. The best K was estimated using cross validation as suggested in the manual.

### 2.5. Phenotypic Traits

We included measurements of phenotypic traits from the same individuals, following the overarching hypothesis that mt haplotype variation may impact phenotypic performances in laying hens.

#### 2.5.1. Measurements of Body Weight, Feed Intake and Phosphorus Utilization

Body weight, feed intake and P utilization were measured for all hens in the first experiment (*n* = 100). Feed intake was calculated over the course of 4 days, by measuring the amount of feed in the beginning and the end of the excreta collection phase that is described in Sommerfeld et al. 2020a [27]. Body weight was measured on the last day of the excreta collection phase. P utilization was calculated as the proportion of intake, which was not recovered in excreta (based on quantitative data, the amount of remaining P in the excreta) for the same periods as feed intake was measured [27]. P utilization was additionally measured for all hens of experiment 2 and analyzed for both experiments separately.

#### 2.5.2. Statistical Analyses

Statistical analysis of body weight and feed intake were performed for all individuals from experiment 1 while the distribution of mt haplotypes over the four diets in the second experiment was heterogeneous, limiting robust statistical tests for these individuals.

The overall impact of the haplotypes on body weight and feed intake was evaluated by a statistical model, derived from a linear mixed model developed by Sommerfeld et al. 2020a [27].

Y = period + haplotype + block + block * period + metabolism unit + father + ε(1)

where Y is the response variable, ε is the residual error, period and haplotype are fixed effects, with block, metabolism unit and father as random effects. Statistical significance was declared at *p* < 0.05.

All modelling was performed in R (R Core Team 2019, Version 3.6.1) using the *lmerTest* package [51]. The interaction of period and haplotype was removed, since the model was rank-deficient due to the absence of one haplotype in period 5. To detect the overall influence of period and haplotype on the response variable, a three factorial analysis of variance (ANOVA) was used. A pairwise Tukey post hoc test (package *emmeans* [52]) was performed to detect differences between the haplotypes independent from the periods and between the periods independent of the haplotype.

To detect significant differences in body weight, feed intake and P utilization between the haplotypes within the periods (and for P utilization within the diets of experiment 2), pairwise Tukey–Kramer HSD or Steel–Dwass tests were performed using JMP Pro (Version 15. SAS Institute Inc., Cary, NC, USA, 1989–2019) after testing for normality using Shapiro–Wilk test.

## 3. Results

### 3.1. Analyses of Mitochondrial Haplotypes

#### 3.1.1. Next Generation Sequencing

The sequencing resulted in an average number of 53,943 reads per DNA sample (min: 36,146, max: 76,180 reads) with a Q30 value of 91.42%. The mapped reads had a mean coverage of approximately 300×, with 0.02% of the nucleotides covered by less than 5 reads.

The validation via mapping the reads against the more distant reference genome resulted in consensus sequences identical to those obtained from the mapping to the white leghorn genome. Further, the Sanger resequencing from individual 23,676 mtDNA and LR-PCR fragments led to identical sequences. Thus, both validation approaches showed that the resulting sequences are not biased by bioinformatics or LR-PCR errors.

#### 3.1.2. Reconstructed Mitochondrial Haplotypes

We reconstructed the mt genomes of 180 individuals and identified 13 segregating sites in the aligned data set. Eight sites are located in nonprotein-coding regions (control region, tRNA-Phe and rRNA) and five sites in protein coding genes (Cytochrome oxidase subunit II (COII), NADH-ubiquinone oxidoreductase subunit 4 and 5 (ND4 and ND5), and Cytochrome B (CytB)) (Table 2). Except one SNP that results in an amino acid change from Serine to Glycine in the ND4 gene (position 12689), all SNPs were silent mutations. The mutations in the control region are located outside of the promoter region (Lan et al. 2015 [1], L’Abbee et al. 1991 [53] for exact positions of this region).

**Figure 2:**
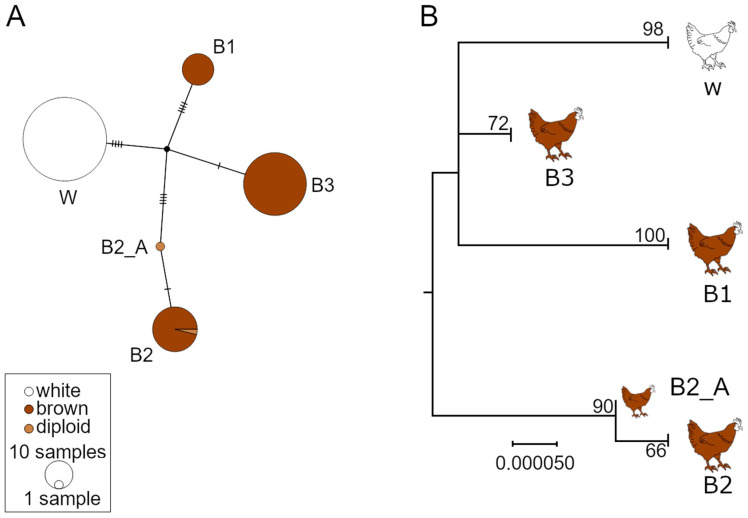
Evolutionary relationship of the mitochondrial genome of laying hens. (**A**) Haplotype network (TCS algorithm) and (**B**) maximum likelihood tree based on the mitochondrial genome (16,784 bp) of 180 laying hens. The tree is based on Hasegawa–Kishino–Yano model with 500 bootstrap replicates and includes all sites.

Four clearly distinct mt haplotypes were discovered: Surprisingly, all individuals of the white strain share the same haplotype whereas the brown strain comprises three haplotypes (Figure 2A, Table 2). There was no variation within each of the single haplotypes, except for one brown individual (B2_A). The B2_A individual appeared to be heterozygote on position 686 in both, the Illumina and Sanger-sequencing approach, and thus appears as a single individual in both trees and haplotype networks (Figure 2).

### 3.2. Haplotype Distribution among Different Periods and Diets

Since the individuals for the experimental phase were chosen randomly out of a bigger group, without previous genotyping, the haplotypes are not equally distributed throughout the five sampling points (experiment 1) or four diets (experiment 2). Only one individual of haplotype B1 occurred in the first period and none occurred in the last period of experiment 1 (Table 3). In the experiment 2 only three individuals of haplotype B1 were included and the number and distribution of haplotype B2 was uneven as well (Table 4).

Equal sample distribution among groups is an essential part of statistical analysis and thus, the unequal distribution and low number of some mt haplotype influences downstream analyses. Nevertheless, the considerable difference between group B1 and B3 in both experiments might reflect the overall population structure of the brown strain.

### 3.3. Nuclear Genotype Data

After filtering for cluster separation, 53,412 SNPs were obtained that were reduced by 1533 SNPs as a consequence of the transfer to the new genome version, resulting in 51,879 SNPs that entered the next filtering steps. During the transfer, all SNP positions changed and 515 SNPs changed the chromosome. Details about remaining SNP numbers per filtering step are given in Supplementary Table S4 for each dataset. Remarkably, a high number of SNPs were identified to be in LD, an observation that is typically found in livestock populations, owing a small effective population size [54]. However, SNPs in LD were only removed for the ADMIXTURE analyses, given by the prerequirements of the algorithm [46], for the calculation of F_ST_ and the G-Matrices these SNPs were included.

#### 3.3.1. Nuclear Diversity between and within the Strains

For the ADMIXTURE analysis of the merged data set (both strains), the algorithm estimated K = 2 as the best number of ancestors. However, K = 3 leads to a separation within the brown strain and rising K to 4 leads to a separation of the white strain, too (Supplementary Figure S1). We calculated a F_ST_ of 0.35 between the two strains, indicating a clear separation of the two strains, too.

**Figure 3:**
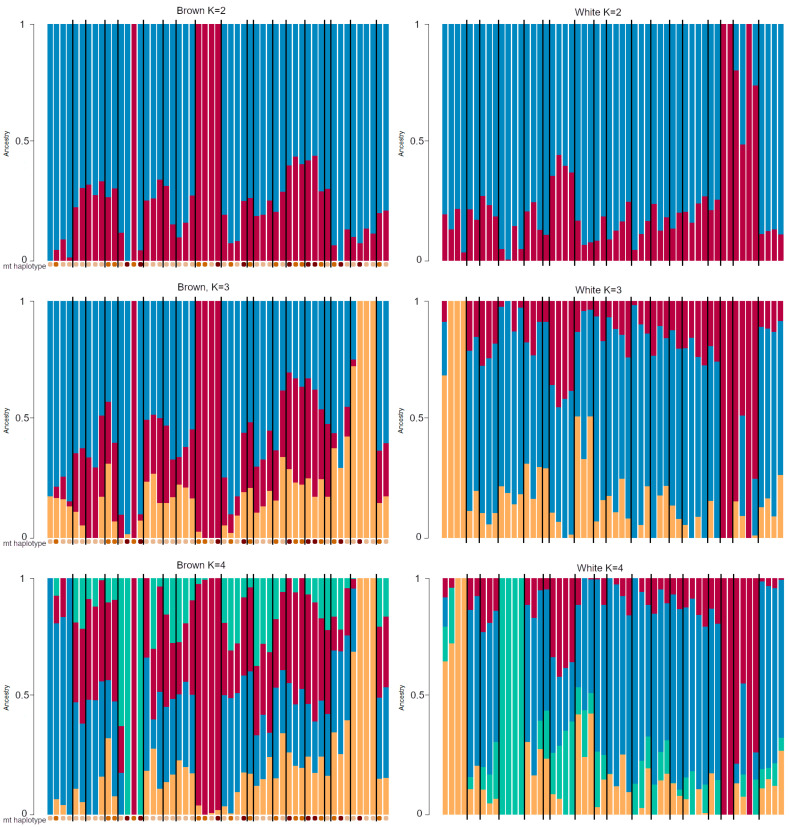
ADMIXTURE plots of the brown and white strain analyzed and filtered separately. Lines separate groups of individuals sharing the same father. Dots under the plots of brown individuals mark the different mt haplotypes.

If strains were separated, the ADMIXTURE analysis estimated 2 to be the best K for both strains. In both strains, some half siblings belong completely to one cluster and this structure does not break down when the number of K is raised, indicating that these individuals are very closely related to each other. The population structure does not seem to be equal to the mt haplotypes, with some brown individuals belonging equally to the same group, independent of the mt haplotype (Figure 3).

#### 3.3.2. Nuclear Diversity between Mitochondrial Haplotypes

The calculations of F_ST_s between the different mt haplotypes of the brown strain were very low (Table 5). The data show that individuals with haplotype B1 are less differentiated from individuals with haplotype B3 on the nuclear level.

#### 3.3.3. Individual Genomic Relationships

**Figure 4:**
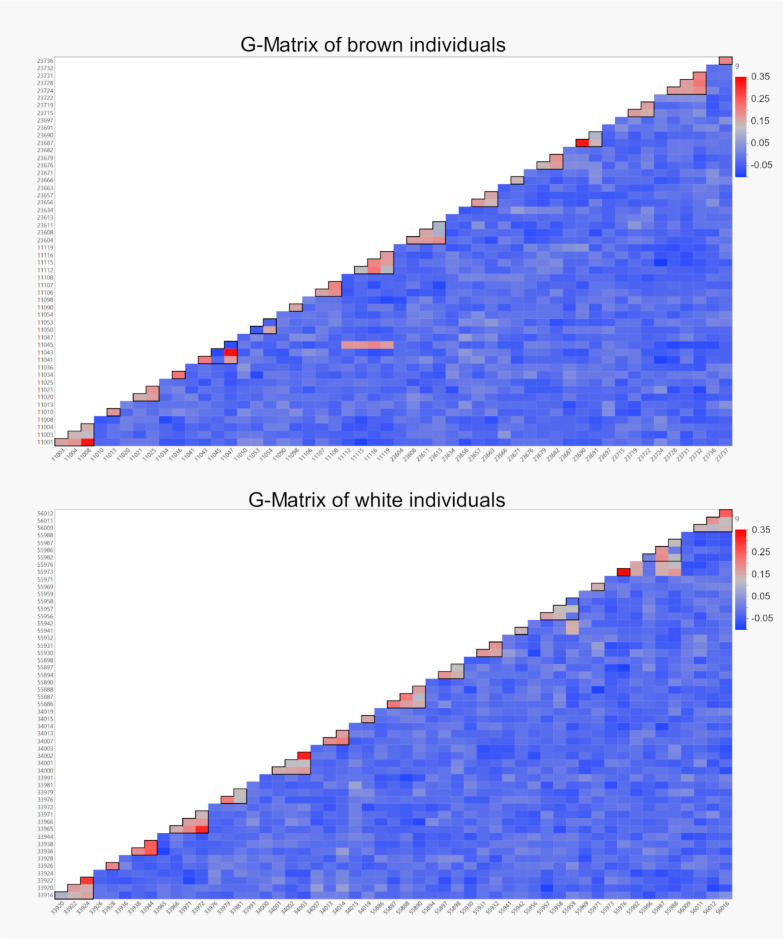
Heat maps of VanRaden G-Matrices for the brown and white individuals. Hens sharing the same father are marked (black lines), individuals are shown in the same order as in the ADMIXTURE plots. The elements of the diagonals are not shown.

The genomic relationship (g) within the two strains did not differ, ranging from −0.1 to 0.34 in the brown and −0.09 to 0.35 in the white strain (Figure 4).

As expected, individuals sharing the same father (half siblings) have a significantly higher genomic relationship than individuals with different fathers (Table 6, Figure 4).

Within the brown strain, individuals sharing the same mt haplotype are closer related than individuals with different mt haplotypes (Table 6), impacted by whether the individuals are half siblings or unrelated. It also became apparent that half siblings were always closer related than individuals with different fathers, independent of if they shared the same mt haplotype or not (*p* < 0.0001 in both cases).

### 3.4. Phenotypic Traits

#### 3.4.1. Phosphorus Utilization

**Figure 5:**
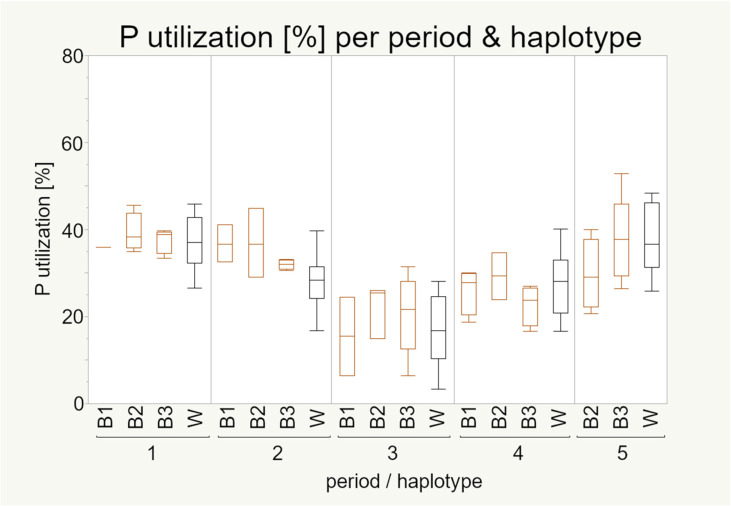
Phosphorus utilization (%) of 100 laying hens by haplotype and period (experiment 1). Boxes represent 50% of the data points (median with interquartile ranges) whiskers show minimum and maximum. Sample numbers are given in Table 3. Statistical significance was declared when *p* < 0.05. P utilization data were first studied by Sommerfeld et al. 2020a [27] in the context of strain differences.

In experiment 1 the P utilization decreased from the first towards the third period, and increased afterwards for all haplotypes (Figure 5). The differences between the highest P utilization (period 1 and 5) to the lowest P utilization (period 3) were significant (Table A2).

The linear mixed model showed that P utilization is not influenced by the mt haplotype but by the period (Table A1). The influence of period was already shown by Sommerfeld et al. 2020a [27]. The pairwise comparison of the haplotypes independent of the period showed no significant difference (Table A2).

The pairwise tests of the haplotypes within each period showed no significant differences as well.

**Figure 6:**
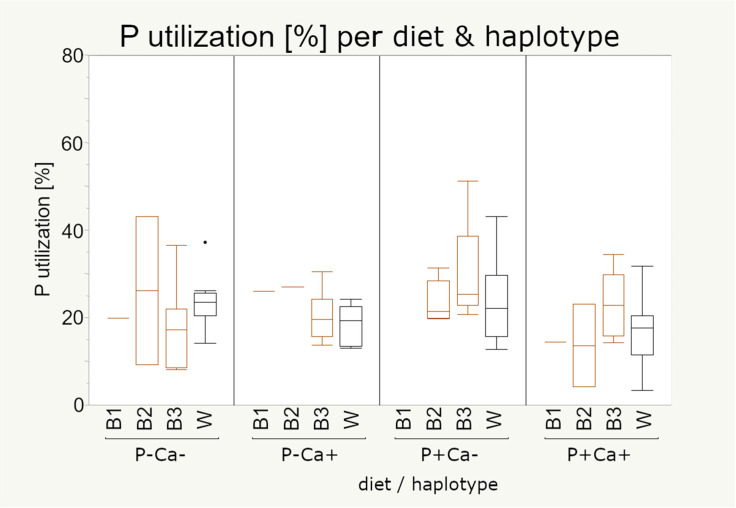
Phosphorus utilization (%) of 80 laying hens by haplotype and diet (experiment 2). Boxes represent 50% of the data points (median with interquartile ranges) whiskers show minimum and maximum. Sample numbers are given in Table 4. Statistical significance was declared when *p* < 0.05. P utilization data were first studied by Sommerfeld et al. 2020b [28] in the context of strain differences.

In the context of different P and Ca concentrations in the diet (experiment 2), it was not possible to observe significant differences between the mt haplotypes, too (Figure 6). However, it became apparent that under high P concentrations individuals of mt haplotype B3 seem to have a higher P utilization than individuals of mt haplotype B2. The most notable observation is the scattering of the brown strain, which is higher than in the white strain under most conditions, even if the number of individuals in the white strain is higher. There were no significant differences between the different diets.

#### 3.4.2. Body Weight

By implementing the multifactorial linear mixed model, there is evidence that the body weight is significantly influenced by both period and mt haplotype (Table A1). However, it must be noted that both strains were analyzed together and that this effect might originate from the high number of white individuals in the dataset rather that the mt haplotypes within the brown strain. Independent of the period, mt haplotype B2 and B3 have a significantly higher body weight than haplotype W, while there was no difference observed between W and B1 (Table A3).

**Figure 7:**
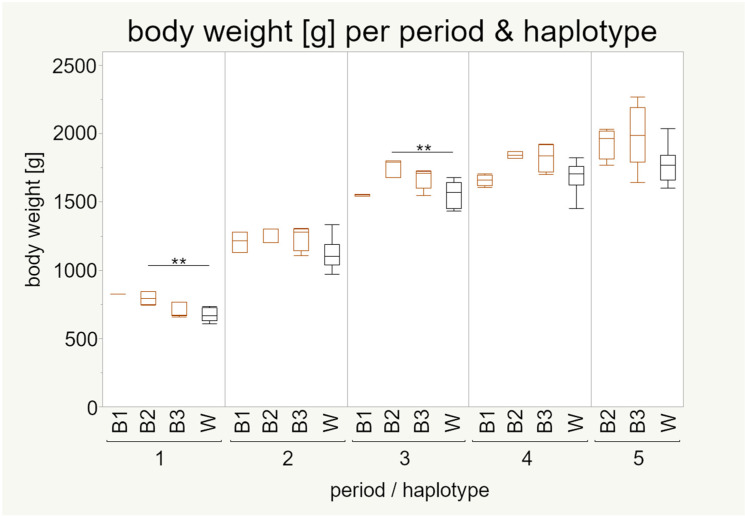
Pairwise Tukey–Kramer HSD or Steel–Dwass tests were used to test for significance within each period. Sample numbers are given in Table 3. Boxes represent 50% of the data points (median with interquartile ranges) whiskers show minimum and maximum. Asterisks indicate significance: ** *p* < 0.01. Body weight was first studied by Sommerfeld et al. 2020a [27] in the context of strain differences.

The body weight increased from period to period for all mt haplotypes and the white strain accumulated less body mass than at least one brown haplotype in all periods (Figure 7). Notably, there was a tendency of lighter individuals carrying B3 haplotype at younger age (period 1, slightly in period 2 and 3) while at the later stages (period 4 and 5), this haplotype showed more variation, including lighter and heavier individuals compared to B2 individuals (Table A4).

Regarding differences between mt haplotypes within the periods, there were no differences except between mt haplotype B2 and W in period 2 and period 3, where individuals of haplotype B2 had a higher body weight (Figure 7). This is a difference between a specific group of brown individuals defined by their mt haplotype with individuals from the white strain, while the body weight of brown individuals with different mt haplotypes is not different to the body weight of the white strain.

#### 3.4.3. Feed Intake

**Figure 8:**
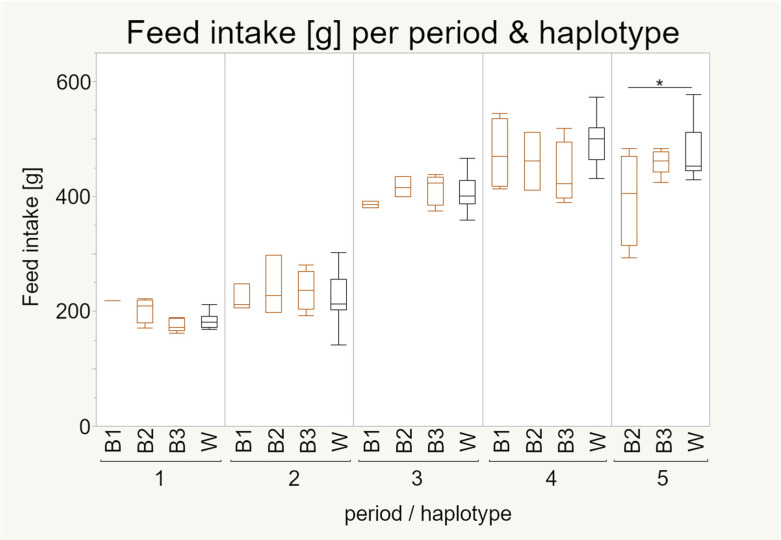
Feed intake (g) of 100 laying hens by haplotype and period. Boxes represent 50% of the data points (median with interquartile ranges) whiskers show minimum and maximum. Pairwise Tukey–Kramer HSD (for normal distributed data) or Steel–Dwass (for nonnormal distributed data) tests were used to test for significance within each period. Sample numbers are given in Table 3. Significance level * = *p* < 0.05. Feed intake was first studied by Sommerfeld et al. 2020a [27] in the context of strain differences.

The model showed that the overall feed intake was significantly influenced by period but not by the different haplotypes (Table A1). In the first two periods, in which the metabolism of the hens was focused on growth [27], the individuals’ feed intake was lower, while in the following three periods (onset and continuation of egg laying), the feed intake was higher (Figure 8). The feed intake differed significantly between all periods, except between period 4 and period 5 (Table A2). In the first and last period the feed intake differed significantly between haplotypes. In the first period the pattern was the same as for body weight (Figure 8) while in the last period, the feed intake of individuals with mt haplotype B2 was significantly less than of the white strain. Additionally, the variance in feed intake in period four was comparably high, which was not reflected in the body weight. Again, these differences reflect the differences between the two strains; nevertheless, they also depict that these strain differences are not present for all haplotypes.

## 4. Discussion

### 4.1. Mitochondrial Haplotypes

The goal of this study was to identify (individual) mt haplotypes and link them to physiological phenotypes.

#### 4.1.1. Low Number of Mt Haplotypes

Our analysis showed that both strains have a surprisingly low number of mt haplotypes: three haplotypes were found in the brown and one in the white strain.

**Figure A1:**
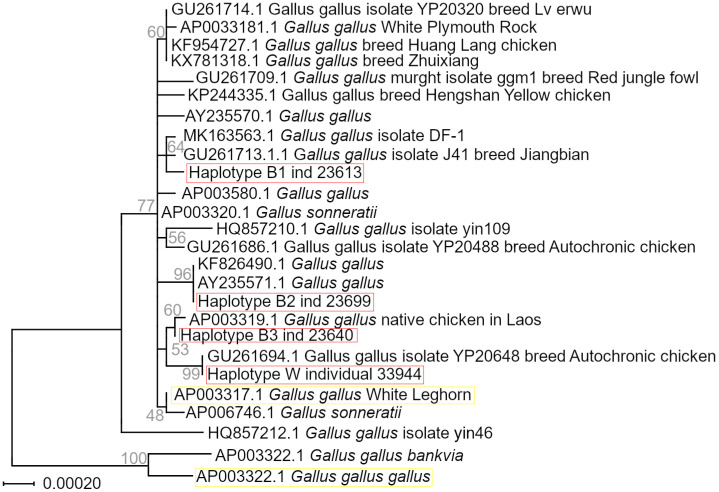
Maximum likelihood tree based on 26 mitochondrial genomes. The tree is based on Hasegawa–Kishino–Yano model including gamma distribution with 200 bootstrap replicates and includes all sites. The alignment was generated with clustalo [81,82], the tree was generated in MEGA X [43]. The tree includes all four detected haplotypes (red), the references used for mapping and validation (yellow) and the five genomes with the highest sequence identity with each haplotype using BLASTn search [83]. Sequences which were under the top five for several haplotypes were only included once and the next highest hits were used to fill up to the final number of five sequences per haplotype.

Other studies showed higher variability in mt haplotypes, but have focused on the highly variable mitochondrial control region (CR). A comparison is possible, since our CR haplotypes are similar to the whole mt haplotypes. Guan et al. 2007 [55] identified two mt haplotypes of CR in 20 white leghorn individuals and Liu et al. 2006 [56] identified nine divergent clades of CR in Eurasian populations. There exist several other studies that have used genetic material from Africa or Asia [1,55,57,58]; however, most likely these individuals were not selected as strongly as the ones in the present study, which would explain the difference in genetic variation. Even though most studies focused on partial mt genomes, there are several complete genomes available such as given by the study of Miao et al. 2013 [59] that includes 60 individuals of different breeds from Asia. Our identified haplotypes cluster well into the published genomes, and the white haplotype is similar to a haplotype identified in Miao et al. 2013 [59]. A maximum likelihood tree with 22 published mt genomes, selected for their similarity to our genomes (via BLAST search) and the references used for the bioinformatics analysis and validation is provided in Appendix B Figure A1.

Due to the maternal inheritance of the mt genomes, our data shows that all white hens can be traced back to one female and the brown hens to three females of origin. This low diversity brought us to question whether the nuclear genetic background is poor due to the breeding history, or if the lack of mt variance is not representative for the whole genetic background of the two strains.

#### 4.1.2. Signs of Heteroplasmy

We observed not only a general low number of mt haplotypes, but also a lack of individual mutations in both strains. Only one individual carrying mt haplotype B2 showed a heterozygous site. Heterozygous appearing sites in the normally haploid mt genome can be a sign of nuclear mitochondrial pseudogenes (numts) [60] or heteroplasmy. Due to our approach amplifying fragments of a size around 9 kb and the low number of known numts in the mt genome of chicken [61], it is rather unlikely that the observed heterozygous site is caused by the sequencing of a numt, supporting heteroplasmy that can be commonly found in mt genomes [62] and has also been observed in chicken [63].

#### 4.1.3. Characterisation of the Identified Haplotypes

The identified haplotypes were characterized by synonymous mutations and mutations in the noncoding mt CR.

Only haplotype B1 contains an amino acid changing mutation in the ND4 gene, which also changes the polarity of the amino acid (serine to glycine). ND4 is part of complex 1 of the respiratory chain and this subunit is not directly part of the electron transport, but anchors complex 1 in the mitochondrial membrane [64]. It is known that mutations in ND4 affect human health [65,66]. In chickens, Li et al. 1998 [9] linked a silent mutation in ND4 with resistance to Marek’s disease, a viral infection affecting the birds’ eyes. The used individuals in our study do not carry this mutation, but the notable impact of a silent mutation in ND4 underlines the potential impact of mutations in this region.

Unfortunately, B1 is the smallest group in our setup, which reduces statistical power. It remains to be elucidated whether the small number of individuals is the result of lower individual performance or rather by coincidence. However, the amino acid change is interesting and needs to be investigated further to gain insights into potential functional effects.

Not only amino acid changing mutations can affect an organism; the influence of synonymous and noncoding mutations has been underestimated for a long time [67]. Now it is known that these mutations can have an impact on, for example, the substrate specificity of the multidrug resistance 1 gene [68] and diseases including cancer [69]. The two main ways silent mutations affect functionality are either through linkage disequilibrium or allele specific differences in mRNA folding and splicing (as reviewed from Kimchi-Sarfaty et al. 2007 and Bali and Bebok 2015) [68,70].

### 4.2. Does the Low Number of Mitochondrial Haplotypes Affect Phenotypic Traits?

None of our included traits is significantly influenced by the mt haplotype—the positive result for body weight is most likely derived from the prominent white strain that has a proven lower body weight [27]. However, our data of the diverse brown strain are more scattered, making an mt haplotype-specific effect more likely. Unfortunately, the unequal sample distribution between the haplotypes in our setup does not allow a robust test for these differences.

The impact of mt haplotypes on body weight is well known in other organisms such as in human [71] or rainbow-trout [72]. This indicates that body weight is a suitable example to demonstrate the importance of the mt genome and its influence on the body (and, thus, for the following experiments) a genotyping previous to the selection of the animals will be included to correct for this and give the analyses more power.

Feed intake and body weight seem to be connected in a way; more feed intake leads to a higher body weight. However, there are some significant differences, since haplotype B2 had a higher feed intake than the white strain in period 5 while not differing in body weight in this time period, but differing in earlier periods with the same feed intake (Figure 7 and Figure 8).

The utilization of P was of the highest interest in our study and, thus, data from both experiments are presented here. In both experiments we were not able to observe significant differences regarding the mt haplotypes but a rather plastic response was found among all groups, especially under the changes of P and Ca concentrations provided in the diets of the second experiment.

However, the link between P utilization and the mt haplotypes is rather complex, which requires additional data from different disciplines such as e.g., from gene expression analyses or metabolites that result from OXPHOS regulatory pathway. The candidates for these analyses are, for example, mt ATPase or ND4, which shows an amino acid change in one of our identified haplotypes, nuclear genes such as AMP-activated protein kinase (AMPK) or Peroxisome proliferator-activated receptor gamma coactivator 1-alpha (PGC1α), which play a role in mitochondrial biogenesis and energy metabolism [73,74]. Myo-inositol as an end product of complete phytate degradation is also discussed to have an impact on mitochondrial biogenesis and function in cell culture cells [75]. As another example, acylcarnitines are indicators of the mitochondrial function with enhanced concentrations in plasma as a consequence of the dysregulation of fatty acid oxidation [76].

Additionally, little is known on how the laying hens and their metabolisms react to the changing dietary conditions related to P bioavailability. Recent studies in broilers have shown an remarkable effect on the plasma metabolome after phytase and myo-inositol supplementation [77].

As a conclusion we were not able to show significant differences between the mt haplotypes but were able to illustrate interesting tendencies that need to be followed up in more stringent experimental design. Furthermore, this would address the current underrepresentation of studies in this field.

### 4.3. Low Mitochondrial Diversity and the Nuclear Genome of the Strains

The observation of little to no mitochondrial variation aroused our interest in the nuclear genome by the usage of the SNP-chip data, enabling insights into the genetic background of the individuals. From the breeding scheme and the way individuals were selected for the experiments (see Figure 1) we know that both strains originate from the same number of fathers and thus, should have a somehow equal genetic background from the paternal side.

From these findings we expected the white strain to be genetically less diverse compared to the brown strain and a general low genetic diversity.

#### 4.3.1. Nuclear Genetic Distance between and Genomic Relatedness within the Strains

Regarding the differentiation between the two lines, the ADMIXTURE analysis showed a clear separation, which was also confirmed by the F_ST_ value (0.35) between them. Gholami et al. 2014 [78] calculated a slightly lower F_STs_ between the three lines of white leghorns derived from Lohmann and a brown layer breed (Rhode Island Red) (F_ST_ = 0.24), but their work includes more lines and SNPs than our work.

To our surprise, the level of genetic relatedness was similar within both strains, giving no indication that the lower mitochondrial diversity of the white strain also exists on the nuclear level. In addition, it became clear in the ADMIXTURE analysis that both strains most likely originated from two lines (K = 2, Figure 3). This leads to an increased number of possible interacting gene products between the mitochondrial and the nuclear genome. The compatibility of nuclear and mitochondrial gene products is known to limit individual fitness [79], which indicates that the higher number of possible combinations can lead to better or worse performing animals. This might explain the often-observed higher variance in measurements (e.g., P utilization) within brown haplotypes, compared to the more similar values within the white haplotype even considering the higher number of white individuals. In addition, the comparable nuclear genomic diversity of the two strains increases the plausibility of effects of the differences in mitochondrial genetic diversity.

#### 4.3.2. Mitochondrial Haplotypes and the Nuclear Genome

The ADMIXTURE analysis showed that some individuals from the brown strain are highly similar independent of their mt haplotype, but often according to their paternal origin (Figure 3). The same individuals appeared as most related on the genomic level in the G-Matrix (Figure 4), which is also a sign that the removal of a high number of SNPs during LD pruning prior to the ADMIXTURE analysis did not disturb the results. The analysis of the population differentiation (F_ST_) confirmed these findings with values close to zero between the three brown haplotypes (Table 5).

Contrasting to these findings, the genomic relationship between individuals with the same mt haplotype is significantly closer than individuals with different mt haplotype (Table 6). However, the effect vanishes when looking only at half siblings. These results show that the maternal genetic background represented by the mt haplotype seems to have a limited role on the genomic relationship compared to the impact of the known father. However, given our experimental design, sharing a mt haplotype does not necessarily imply the same nuclear genomic background provided by the mother. Thus, it becomes obvious that individuals with the same mt haplotype are not as closely related as individuals with the same father, providing potential for modifications in mito-nuclear interactions [80].

## 5. Conclusions

Linking mt haplotypes and phenotypic traits is of high interest but was not fully possible in this study. Nevertheless, the rather surprisingly low mitochondrial diversity is still interesting, given the contrasting and high-yielding performance of these two strains. In addition, this study examined a part of the genome that is normally not used in breeding approaches, and even if the mt genome does not seem to be representative for the whole genetic background, the low diversity is worrying and provides important information about the breeding history of the strains. However, further analyses including the determination of the nuclear genetic background from the maternal side might lead to a better understanding of the mito-nuclear interaction, an interesting and so far, less explored topic in the breeding of laying hens.

## Figures and Tables

**Table 1:** P and Ca concentrations in the four diets of experiment 2. Values from Table 1 in Sommerfeld et al. 2020 [28]. Recommended concentrations are labelled with +, reduced concentrations with **−**.

Ingredient g/kg	P−Ca−	P−Ca+	P+Ca−	P+Ca+
**Total P, g/kg DM ^1^**	4.7	4.7	5.3	5.3
**Ca, g/kg DM ^1^**	33.9	39.6	33.9	39.6

^1^ Dry mass.

**Table 2:** Overview of the Single Nucleotide Polymorphism (SNPs) of the deduced four haplotypes with number of individuals (*n*), gene/region, position and corresponding nucleotide.

Haplotype		B1	B2	B2_A	B3	W
*n*		13	26	1	51	90
**Gene**	**Position**					
Control Region	199	C	T	T	T	T
	222	A	G	G	A	A
	243	C	C	C	C	T
	256	T	C	C	C	C
	330	C	T	C	C	C
	342	A	A	G	G	A
	686	G	G	A	A	A
	859					C insertion
tRNA^Phe^	1297	T	C	C	T	T
rRNA	1526					A insertion
COII	8788	C	C	C	C	T
ND4	12013	T	T	T	T	C
	12689	A	G	G	G	G
ND5	13232	A	G	G	A	A
CytB	15840	T	T	T	T	C

Abbreviations: rRNA (Ribosomal ribonucleic acid), COII (Cytochrome oxidase subunit II), ND4 and ND5 (NADH-ubiquinone oxidoreductase subunit 4 and 5), CytB (Cytochrome B).

**Table 3:** Number of individuals per haplotype and period obtained from experiment 1.

Haplotype/Period	1	2	3	4	5
B1	1	3	2	4	0
B2	4	3	3	2	4
B3	5	4	5	4	6
W	10	10	10	10	9

**Table 4:** Number of individuals per haplotype and diet obtained from experiment 2.

Haplotype/Diet	1	2	3	4
B1	1	1	0	1
B2	2	2	5	1
B3	7	7	5	8
W	10	10	10	10

**Table 5:** F_ST_ values calculated between individuals with different mt haplotypes.

Haplotype Groups	F_ST_
B1 vs. B2	0.00035
B1 vs. B3	0.0044
B2 vs. B3	0.0045

**Table 6:** Means of g and *p*-values of compared groups with different haplotype and relationship statuses.

Group 1	Group 2	Mean Group 1	Mean Group 2	*p*
Not related both strains	Half sibling both strains	−0.02	0.16	<0.0001
Not related white	Half sibling white	−0.02	0.16	<0.0001
Not related brown	Half sibling brown	−0.02	0.15	<0.0001
The following only tested in the brown strain				
Same haplotype	Diff. haplotype	−0.0086	−0.0016	0.008
Unrelated same hapl.	Unrelated diff. hapl.	−0.017	−0.022	0.0069
Half sibling same hapl.	Half sibling diff. hapl.	0.137	0.138	ns

## Data Availability

All sequencing data obtained in this study were deposited on GenBank under the accession numbers MT800324–MT800504.

## Supplementary Materials

The following are available online at https://www.mdpi.com/2076-2615/11/3/825/s1: Table S1: Primer used for resequencing and validation with position, length and according long-range fragment; Table S2: PCR conditions used for all PCR reactions.; Table S3: PCR conditions used for all PCR reactions; Figure S1: ADMIXTURE plots of the brown and white strain analyzed and filtered together; Table S4: SNPs left after filtering for all three datasets, filters were applied one after another from top to bottom.

## Appendix A

**Table A1:** Influence of period and haplotype on body weight, feed intake and phosphorus utilization. *p*-values are from the evaluation of the statistical model using a three factorial anova.

Response Variable	Body Weight	Feed Intake	P Utilization
Period	<2.2 × 10^−16^	<2 × 10^−16^	5.965 × 10^−11^
Haplotype	4.925 × 10^−5^	ns ^1^	ns

^1^ not significant.

**Table A2:** *p*-values of pairwise comparisons of body weight, feed intake and phosphorus utilization between periods independent of the haplotype. Tukey post hoc was used for testing.

Difference	Body Weight	Feed intake	P Utilization
1 vs. 2	<0.0001	0.0094	ns
1 vs. 3	<0.0001	<0.0001	<0.0001
1 vs. 4	<0.0001	<0.0001	0.0002
1 vs. 5	<0.0001	<0.0001	ns
2 vs. 3	<0.0001	<0.0001	<0.0001
2 vs. 4	<0.0001	<0.0001	ns
2 vs. 5	<0.0001	<0.0001	ns
3 vs. 4	0.0118	<0.0001	ns
3 vs. 5	<0.0001	0.0016	<0.0001
4 vs. 5	0.0123	ns	0.0012

**Table A3:** *p*-values of pairwise comparisons of body weight, feed intake and phosphorus utilization between haplotypes independent of the period. Tukey post hoc was used for testing.

Haplotype	Body Weight	Feed Intake	P Utilization
B1 vs. B2	0.0581	ns	ns
B1 vs. B3	ns	ns	ns
B1 vs. W	ns	ns	ns
B2 vs. B3	ns	ns	ns
W vs. B2	0.0007	ns	ns
W vs. B3	0.0009	ns	ns

**Table A4:** *p*-values of the comparison of body weight of the different haplotypes per period. Pairwise Tukey–Kramer HSD or Steel–Dwass tests were used for testing depending on the distribution of the data.

Haplotype/Period	1	2	3	4	5
B1 vs. B2	ns	ns	ns	ns	- ^1^
B1 vs. B3	ns	ns	ns	ns	-
B1 vs. W	ns	ns	ns	ns	-
B2 vs. B3	ns	ns	ns	ns	ns
W vs. B2	0.0045	ns	0.0067	ns	ns
W vs. B3	ns	ns	ns	ns	ns

^1^ no statistical test performed, no animals of group B1 present.
