# Supplementary material for: Insights into the Mitochondrial and Nuclear Genome Diversity of Two High Yielding Strains of Laying Hens
Source: Animals (Basel). 2021 Mar 15;11(3):825. doi: 10.3390/ani11030825 (PMC8001891; doi:10.3390/ani11030825)
Supplement: Supplementary file 1 [file animals-11-00825-s001.zip › SupplementaryTables.docx]

**Table S1.** **Primer used for re-sequencing and validation with position, length and according long-range fragment**. Positions are according to the used reference genome (Accession: AP003317).

| **Primer** | **Position** | **Length [bp]** | **Sequence** | **LR-Fragment** |
| --- | --- | --- | --- | --- |
| 1F  1R | 16606  721 | 923 | ATCACAGACGCTACCACCAA  CAGGTGTAGTCCAGGCTTCA | Fragment 1 |
| 2F  2R | 502  1361 | 879 | CCTACTTGCCTTCCACCGTA  GGGGCATTTTCACTGGGATG |  |
| 3F  3R | 1248  2133 | 905 | CCACAAAGCATGGCACTGAA  TGCCTCAGAGCCGTCTTAAA |  |
| 4F  4R | 1913  2873 | 980 | TTGCCAGCACAGCCTACATA  GCGGGGAGGGGTGATTATTA |  |
| 5F  5R | 2769  3686 | 937 | CCCCTAAAGACACCCACCTT  CCTCCTTGTCGATATGGGCT |  |
| 6F  6R | 3657  4504 | 867 | CCAGGGATAACAGCGCAATC  GATGGCTAGGGTGACTTCGT |  |
| 7F  7R | 4483  5373 | 910 | CCGTCGCACAAACAATCTCA  TGTGACTTGGAGATGAGGGG |  |
| 8F  8R | 5299  6245 | 973 | CCAGCATCACAATCTCCAGC  AAAGGGGCAATAGGGTGGTT |  |
| 9F  9R | 5986  6872 | 906 | TAACCCTCCTCTCCCTAGCT  CGAAGCCACCGATCATGATG |  |
| 10F  10R | 6781  7695 | 934 | GACAGCCCGGAACTCTCTTA  AGGCCTCCGATAGTGAAGAG |  |
| 11F  11R | 7555  8529 | 994 | ACACCCGAGCCTACTTTACA  GCGGGTAGGATGGTTCAGAT |  |
| 12F  12R | 8500  9361 | 881 | ACACCGTAGATGCCCAAGAA  GAGGCGGTTGTTGATCCATC |  |
| 13F  13R | 9317  10161 | 895 | ATCACTCCTTCTTCCAGCCC  TCCGTATCGTAGGCCCTTTT | Fragment 2 |
| 14F  14R | 10095  11049 | 974 | TGCTGCAATGATGACGAGAC  TGGTGGCTCAAGTAAGGGTT |  |
| 15F  15R | 10988  11912 | 944 | ACCTAGAAATCGCCCTGCTT  CGCTAAGTCGTTCTGGTTGG |  |
| 16F  16R | 11863  12703 | 860 | TCGAAGCAACCCTAATCCCA  GAGGGTGTAAGAGGCGGTTA |  |
| 17F  17R | 12542  13448 | 926 | CCAACCCCTCCTACCATTGA  GGCGATGAGGAAGGTGAGTA |  |
| 18F  18R | 13298  14199 | 921 | TCCCCAACTTCAAGATCCCC  CGAGGTTGCCAATGGTTAGG |  |
| 19F  19R | 14049  15026 | 997 | CCAACTAGCCTTCCTCCACA  AGGATTTGGGTCATGAGGCA |  |
| 20F  20R | 14907  15793 | 852 | TGGCACCCAACATTCGAAAA  TGGAGCGTAGGATGGCATAG |  |
| 21F  21R | 15665  16587 | 942 | CCAGAAAACTTCACCCCAGC  TAGGATGCGGGTGGTTAGTG |  |
| 22F  22R | 16488  375 | 659 | AGGATCCGCAGCTAATGACA  ACGGCGAGCATAACCAAATG | Fragment 1 |

**Table S2.** **PCR conditions used for all PCR reactions.** Dream Taq (Thermo Fisher scientific Inc., Massachusetts, USA) was used according to the manufacturers manual.

| Temperature [°C] | Time |  |
| --- | --- | --- |
| 95 | 2 min |  |
| 95 | 20 s | 35× |
| 54 | 15 s |  |
| 72 | 45 s |  |
| 72 | 2 min |  |
| 4 | ∞ |  |

**Table S3.** **PCR conditions used for all PCR reactions.** Dream Taq (Thermo Fisher scientific Inc., Massachusetts, USA) was used according to the manufacturers manual.

| sample | re-sequenced parts or used primers |
| --- | --- |
| 11045 | Fragment 1 |
| 11050 | Fragment 1 |
| 23635 | Fragment 1 |
| 23715 | Fragment 1 +22 |
| 11020 | Fragment 2 |
| 34003 | Fragment 2 |
| 55935 | Fragment 2 |
| 55940 | Fragment 2 |
| 55957 | Fragment 2 |
| 56009 | Fragment 2+ 1 |
| 11019 | 1, 2 |
| 11099 | 1,16-22 |
| 23671 | 1 |
| 23699 | 21 |
| 23731 | 2 |
| 34007 | 18 |
| 55976 | 8 |
| 55986 | 21 |
| 55987 | 8 |
