# Supplementary figures and images for: Insights into the Mitochondrial and Nuclear Genome Diversity of Two High Yielding Strains of Laying Hens
Source: Animals (Basel). 2021 Mar 15;11(3):825. doi: 10.3390/ani11030825 (PMC8001891; doi:10.3390/ani11030825)

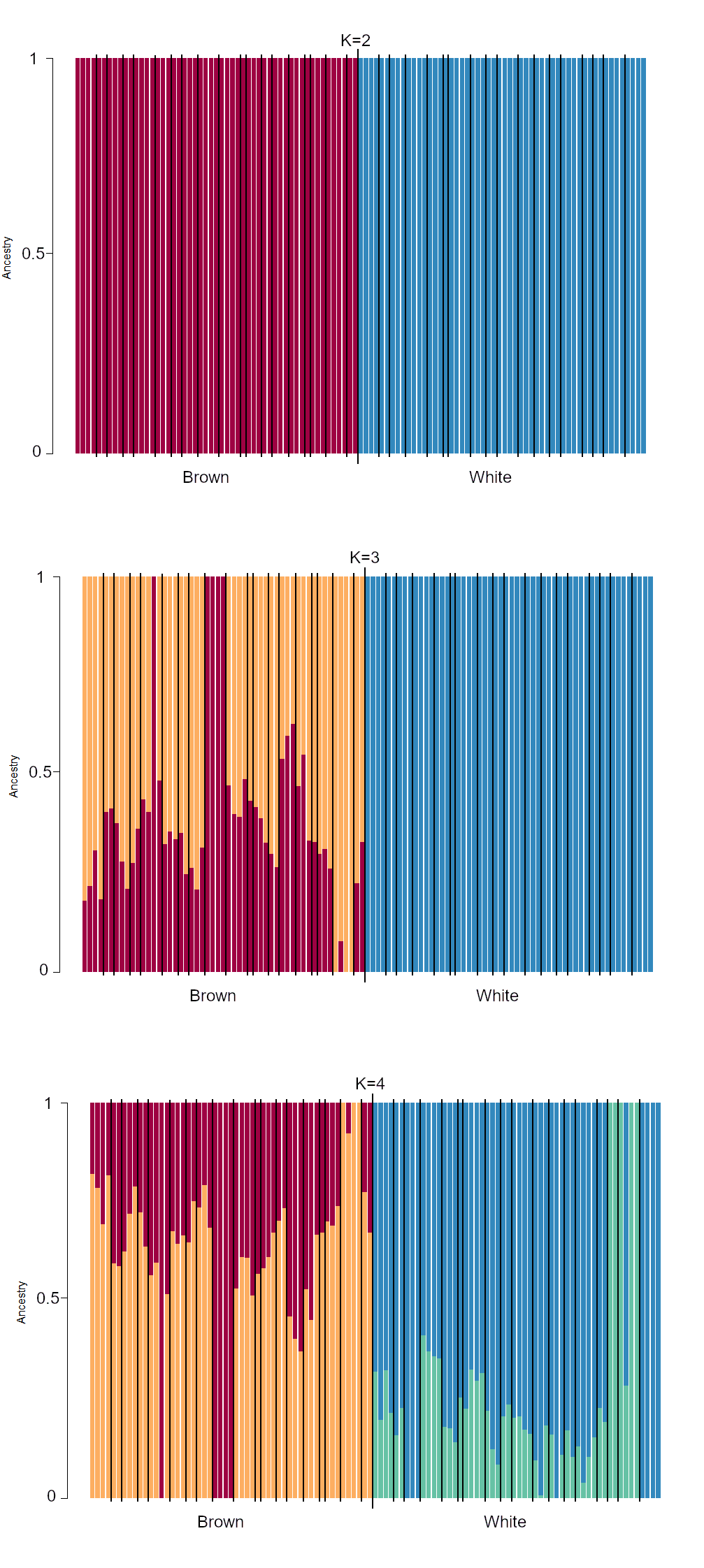

Supplement: Supplementary file 1 [file animals-11-00825-s001.zip › SupplementaryFigureS1.png]
